# Optimization of Biocatalytic Rhododendrol Production from Biogenic Rhododendrol Glycosides

**DOI:** 10.1021/acssuschemeng.4c05889

**Published:** 2024-10-18

**Authors:** Emerik Leaković, Karsten Siems, Michel Feussi Tala, Antonia Habazin, Zvjezdana Findrik Blažević, Ana Vrsalović Presečki

**Affiliations:** † University of Zagreb Faculty of Chemical Engineering and Technology, Trg Marka Marulića 19, HR-10000 Zagreb, Croatia; ‡ AnalytiCon Discovery GmbH, Hermannswerder 17, 14473 Potsdam, Germany

**Keywords:** betuloside, apiosylrhododendrin, enzymatic hydrolysis, β-glucosidase, RAPIDASE, mathematical modeling

## Abstract

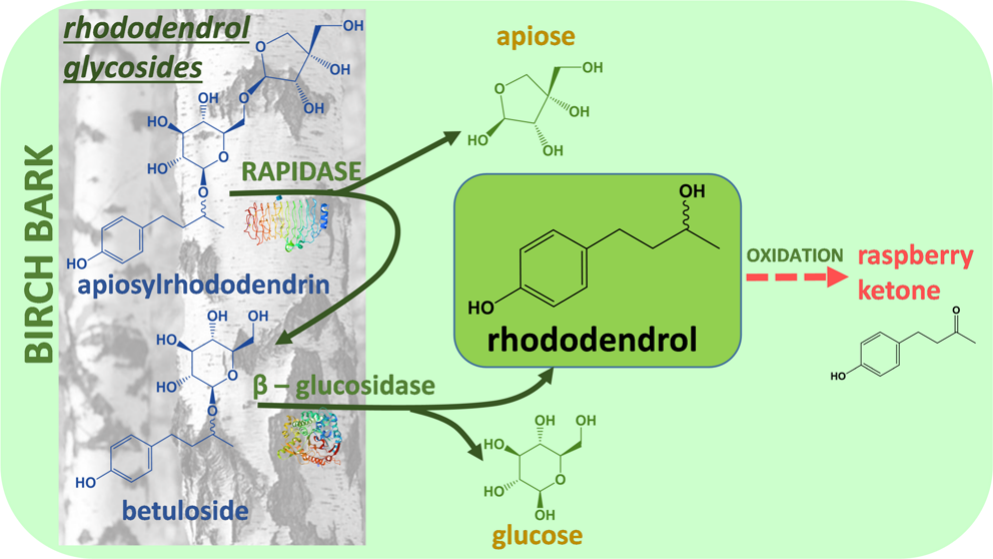

An enzyme-catalyzed synthesis of rhododendrol, an intermediate in the production of raspberry ketone, was investigated. The approach involves the enzymatic hydrolysis of rhododendrol glycosides into rhododendrol and a glycosidic residue. Rhododendrol glycosides, which are naturally derived from the inner bark of birch trees—a renewable resource—vary considerably in composition depending on the origin of the plants. In this study, mixtures of betuloside and apiosylrhododendrin from natural resources were used in different proportions. An in-depth study was conducted to assess the feasibility of the process. A mathematical model was developed based on studies of the kinetics and operational stability of the enzyme. The model for betuloside hydrolysis catalyzed by β-glucosidase was validated in batch, repetitive batch, and ultrafiltration membrane reactors. The highest productivity, ranging from 83.9 to 94.5 g L^–1^ day^–1^, was achieved in the latter. After screening nearly 50 enzymes, RAPIDASE emerged as a solution for the hydrolysis of apiosylrhododendrin, and the model was validated in a batch reactor. Model-based optimization enabled the prediction of input parameters for different compositions of biogenic rhododendrol glycosides to obtain consistent process output metrics.

## Abstract 2

Model-based optimization enabled the utilization of birch bark residues for the sustainable synthesis of rhododendrol, a precursor of raspberry ketone.

## Introduction

Nutraceuticals are substances that are used as dietary supplements but can also be used as pharmaceuticals. The term nutraceutical was coined by Stephen Defelice in 1989, combining the terms nutrient and pharmaceutical. The main application of nutraceuticals is in nonspecific biological therapies, e.g., for general well-being of the organism, control of disease symptoms and metabolic disorders, and prevention of malignant processes.^1,2^ An example of a nutraceutical obtained from natural renewable sources (plant origin) is raspberry ketone. Raspberry ketone is a natural phenolic compound that is the primary aromatic compound of red raspberry. It is used as a flavoring agent in the food industry and as a nutraceutical, e.g., dietary supplement for weight regulation.^3^ It has been shown to have liver and lung protection properties,^4,5^ affect skin elasticity, and promote hair growth, and it also has antioxidant properties.^6,7^ One way to obtain raspberry ketone is to isolate it directly from the fruit. However, the content of the compound in the fruit is very low (1–4 mg/kg fruit), making this method of isolation economically unviable.^8^ The traditional approach for the synthesis of raspberry ketones is chemical synthesis. Although this is a fairly efficient and cost-effective approach, it usually results in products that cannot be labeled as “natural” and therefore cannot be readily offered to different markets and sectors.^9−11^ Several microorganisms were developed for this purpose. The efficiency of raspberry ketone production in this way is low and undesirable side reactions occur due to the presence of other enzymes in the cell.^12−14^ Another possibility for obtaining raspberry ketone is a biocatalytic method. Enzymatic synthesis is an environmentally friendly alternative to chemical synthesis and offers the possibility of obtaining high yields. One of the methods of enzymatic synthesis of raspberry ketone is the one that mimics the metabolic pathway of synthesis in the plant itself.^15,16^ Another pathway is the use of cytochrome P450 BM3, which can catalyze the hydroxylation of 4-phenyl-2-butanone, a natural precursor, to raspberry ketone.^17^ The third option includes the biocatalytic transformation of the starting material obtained from birch bark, a renewable substrate source that has a high content of rhododendrol glycosides. Birch bark is a byproduct of paper and furniture industry, which makes this process sustainable. These glycosides are the substrates in an enzymatic process that involves two steps. The first step is hydrolysis, i.e., separation of the rhododendrol from the glycoside moiety using the hydrolase. Then, the alcohol obtained is oxidized to the desired raspberry ketone by the action of oxidoreductase, i.e., NAD(P)-dependent alcohol dehydrogenase, together with coenzyme regeneration.^18^ NAD(P) stands for nicotinamide adenine dinucleotide (phosphate).

To develop an effective and feasible biocatalytic process, examining the kinetic properties of enzymes is crucial. Assessing a kinetic model early in the process development stage can be highly beneficial for subsequent implementation. It facilitates evidence-based decision-making, helps identify bottlenecks, and quantifies potential process issues such as feedback inhibition or inhibition by side-products.^19^ Effective process scale-up can be achieved through miniaturized experiments or mathematical modeling. Mathematical models enable fast and cost-effective reaction optimization. The true value of such models lies in their ability to predict, evaluate, and explore various scenarios or assumptions within the modeled system and its environment, including changes in initial reaction conditions and variations in reagent and/or catalyst concentrations.^20,21^

**Figure 1:**
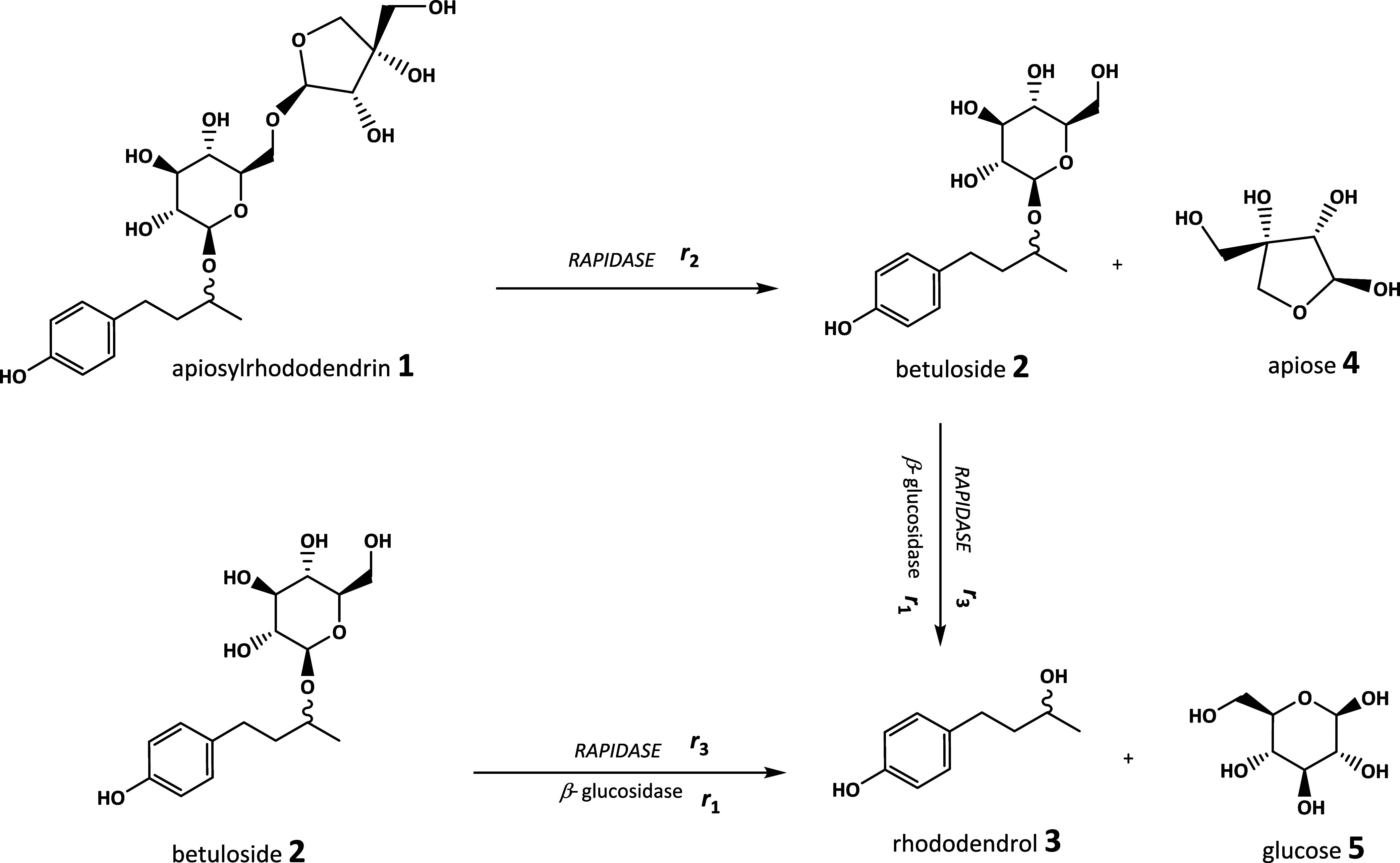
Reaction of rhododendrol glycoside hydrolysis catalyzed by β-glucosidase and RAPIDASE.

This study deals with the first step in the production of raspberry ketone from renewable sources (birch bark), i.e., the hydrolysis of rhododendrol glycosides. There are two types of rhododendrol glycosides found in the inner bark of *Betula pubescens*: betuloside and apiosylrhododendrin. The proportion of betuloside and apiosylrhododendrin in the mixture of rhododendrol glycosides can vary considerably after extraction from the birch bark. Betuloside can be hydrolyzed by β-glucosidase to rhododendrol (precursor in the production of raspberry ketone) and glucose (Figure 1). It was found that apiosylrhododendrin can be hydrolyzed to betuloside and apiose by the commercial enzyme preparation RAPIDSE.^22^ Based on independent kinetic and/or stability studies, a mathematical model was created and validated for the different reactor types. The literature shows that there are no detailed studies of the optimization of this process. The results published so far on this system include the results of Becker et al., which were done on a much smaller scale.^18^ They used 10 mg mL^–1^ rhododendrol glycoside mixture and achieved conversion of 80 ± 1% within 2 h using 1 mg mL^–1^ β-glucosidase from almonds. The objective of this work was to optimize the enzymatic hydrolysis of rhododendrol glycosides to produce rhododendrol, which will then serve as a substrate for the second stage of the sequential production of raspberry ketones, eliminating the need for intermediate product purification.

## Experimental Section

### Chemicals

Dipotassium phosphate and monopotassium phosphate were purchased from VWR Chemicals. Sodium acetate, disodium carbonate, glucose, potassium sodium tartrate tetrahydrate, sodium hydroxide, and 3,5-dinitrosalicylic acid (DNS) were purchased from Gram-Mol (Croatia). Apiose was purchased from Biosynth (Switzerland). *p*-Nitrophenyl-β-d-glucopiranoside (p-NPG) and acetonitrile were provided by Fisher Scientific. Polygalacturonic acid (PGA) and trifluoroacetic acid (TFA) were obtained from Sigma-Aldrich. Rhododendrol (racemic mixture) was purchased from TCI (Japan). The rhododendrol glycosides, used as substrates in the reactions, were provided by AnalytiCon Discovery GmbH (Germany) and are a mixture of betuloside and apiosylrhododendrin in various ratios (mixture 1: ω_betuloside_ = 74.0%, ω_apiosylrhododendrin_ = 26.0%, mixture 2: ω_betuloside_ = 27.5%, ω_apiosylrhododendrin_ = 72.5%).

β-Glucosidase from almonds was purchased from Sigma-Aldrich. RAPIDASE (REVELATION AROMA) was purchased from DSM Oenobrands (France) and is a mixture of β-glucosidase and polygalacturonase (pectinase).

### HPLC Measurements

The concentrations of rhododendrol glycosides and rhododendrol (offline concentration monitoring) during the reaction were measured by using the high-performance liquid chromatography (HPLC) method. The analysis was performed at 30 °C on a Phenomenex Kinetex Reversed Phase C18 (250 × 4.6 mm^2^, 5 μm) column at a flow rate of 1.5 mL min^–1^ using the gradient method. The mobile phase A was a mixture of acetonitrile, water, and trifluoroacetic acid (TFA) in a ratio of 80:20:1 (v/v/v); the mobile phase B was water acidified by TFA (0.1% v/v%). The gradient was adjusted to 90–40.7324% for the first 8 min and to 40.7324–90% from 8 to 10 min. Detection was performed at 200 nm. The retention time of apiosylrhododendrin and betuloside was 4.8 and 5.0 min, respectively, while the retention time of rhododendrol was 6.3 min. An example of a chromatogram of the analyzed chemicals and calibration curves for all three compounds are shown in Figures S1 and S3.

### Activity and Stability of Enzymes

The activity and stability of the enzymes in potassium phosphate buffer were investigated at different pH values and at different temperatures. The operational stability was also monitored for each reaction. Operational stability was described as the relative activity of the enzyme during its use in a biocatalytic process in comparison with the activity of the enzyme at the beginning of the process.

The specific activity of the enzymes in the studied reactions (Figure 1) was examined by starting the hydrolysis with the mixture of betuloside or apiosylrhododendrin as a substrate at different pH values and temperatures. In these measurements, the rhododendrol concentrations were monitored by HPLC, and the collected data was used to estimate the initial reaction rate.

To determine the stability of the enzyme under different conditions (pH and temperature) and to follow its operational stability during a reaction, a spectrophotometric activity assay was performed by using more available and preferably cheaper substrates. The operational stability is monitored at specific time intervals. Five samples (30 μL) were taken from the reactor, and the enzyme was filtered by centrifugal filter units (CFUs) (14,000 rpm, 4 °C, 15 min). Enzyme remaining in the CFU was resuspended in 60 μL of ultrapure water. With this resuspended enzyme, activity assays were run. Spectrophotometric assays for both enzymes are described in the following two paragraphs.

β-Glucosidase activity assay is based on the reaction with *p*-nitrophenyl-β-d-galactopiranoside (*p*-NPG) as substrate. The product of the catalytic reaction is *p*-nitrophenol (*p*-NP), which absorbs light at 405 nm.^23^ Reaction scheme can be found in the Supporting Information (Figure S14). 10 μL of resuspended enzyme (after filtration) was mixed with 390 μL of 50 mM sodium acetate buffer pH 5.5 and 100 μL of 10 mM p-NPG. The mixture was incubated at 50 °C for 10 min. After 10 min, 500 μL of 128 mM sodium carbonate was added to stop the reaction and the absorbance was determined at 405 nm.

RAPIDASE is a mixture of β-glucosidase and polygalacturonase (pectinase). The determination of polygalacturonase activity is based on the reaction of hydrolysis of polygalacturonic acid (PGA).^24^ The amount of the product of the catalytic reaction can be quantified by DNS test. 5 μL of resuspended enzyme was mixed with 45 μL of 5 mg mL^–1^ polygalacturonic acid. The mixture was incubated for 15 min at 40 °C and 900 rpm. After 15 min, 100 μL of DNS reagent (Section S3) was added, followed by a further 5 min incubation at 100 °C without mixing.^25^ To stop the reaction, 1 mL of ultrapure water was added, and the absorbance was determined at 540 nm. Galacturonic acid reduces 3,5-dinitrosalicylic acid to 3-amino-5-nitrosalicylic acid, which absorbs light at 540 nm. The reaction scheme is shown in the Supporting Information (Figure S15). This test with PGA and DNS reagent is not standardized.^24,25^ For this reason, the test was validated. Validation of the PGA-DNS test is explained in the Supporting Information in more detail (Section S4).

### Enzyme Kinetics

The kinetic parameters of hydrolysis of betuloside by β-glucosidase from almonds and apiosylrhododendrin by RAPIDASE (both β-glucosidase and polygalacturonase) were determined using the initial reaction rate method. The initial reaction rate was measured at a substrate conversion below 10%. The influence of the substrate concentration on the initial reaction rate enables estimation of the kinetic parameters. The influence of the concentration of products on the reaction was also investigated to assess the presence of product inhibition. For this analysis, the concentration of the substrate was kept at a saturating level, while the concentration of the product was varied. The reaction rate was determined by HPLC by following the production of rhododendrol for β-glucosidase and the consumption of apiosylrhododendrin for polygalacturonase. The kinetics were determined at the optimal conditions for this reaction: 0.1 M phosphate buffer pH 6, 40 °C. The reactions are irreversible, and the kinetic parameters were therefore only estimated for the hydrolysis reaction.

### Reactor Experiments

To validate the developed mathematical models and the estimated kinetic parameters, the reaction of betuloside hydrolysis catalyzed by β-glucosidase from almonds was carried out in three reactor types: batch reactor (BR), repetitive batch reactor (RBR), and ultrafiltration membrane reactor (UFMR, Bioengineering AG, Switzerland). All reactions were done in 0.1 M phosphate buffer pH 6 at 40 °C with the enzyme concentration of 4 mg mL^–1^. The volume of the BR was 2 mL with a betuloside concentration of 275 mM. The BR was shaken with a shaker at 900 rpm. The RBR with a reaction volume of 2 mL was conducted in a microtube, which was used as a BR and placed on a shaker at 900 rpm. The reaction was carried out with a total of 5 substrate additions (60 mM per addition). New amounts of betuloside were added as soon as complete conversion was achieved. The UFMR volume was 10 mL with a cutoff membrane of 10 kDa (Ultracel Amicon YM 10). The betuloside concentration was 62.3 mM at a flow rate of 180 μL min^–1^. Compared to the reactions carried out in the batch reactor, a lower betuloside concentration was chosen due to the viscosity of the rhododendrol glycoside solution, which could significantly increase the pressure in the membrane reactor and potentially negatively affect the stability of the enzyme. The continuous flow into the reactor was maintained constant and uniform with a piston pump (RCT M160, Heidelberg, Germany).

RAPIDASE was used for the hydrolysis of apiosylrhododendrin. Apiosylrhododendrin is hydrolyzed to betuloside and apiose by polygalacturonase from the enzyme mixture, followed by betuloside hydrolysis to rhododendrol and glucose by β-glucosidase (Figure 1). Besides by β-glucosidase from almonds, betuloside can be hydrolyzed by β-glucosidase present in the RAPIDASE mixture. For that reason, to validate the developed mathematical model and the estimated kinetic parameters, a total of four reactions were carried out in a batch reactor (BR): 1. RAPIDASE + batch of rhododendrol glycosides with a higher apiosylrhododendrin ratio, 2. RAPIDASE + rhododendrol glycosides with a higher betuloside ratio, 3. RAPIDASE + β-glucosidase from almonds + batch of rhododendrol glycosides with a higher apiosylrhododendrin ratio, 4. RAPIDASE and β-glucosidase from almonds using a batch of rhododendrol glycosides with a higher betuloside ratio. The detailed reaction conditions are given in the descriptions in Figure 3.

Samples were taken at specific time intervals during all reactions to monitor the substrate and product concentrations and determine the operational stability of the enzymes. The total amount taken from the reactor was less than 10% of the volume of the reaction mixture.

### Data Processing

The kinetic parameters and enzyme inactivation constants of the proposed models were estimated by nonlinear regression analysis, i.e., least-squares method using the simplex or Powell algorithms implemented in the MicroMath Scientist software (SCIENTIST, 1986–1995). For the simulations, the built-in episode algorithm was used to solve the system of differential equations.

Standard deviations (σ, eq 1) and coefficient of determination (*R*^2^, eq 2) of the goodness-of-fit curve were provided also by Scientist, where *n* is the number of points, *Y*_obs_ is the experimental value, and *Y*_cal_ is the value calculated by the model.
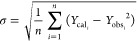
1
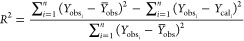
2

Additionally, three process metrics were calculated from the data collected from the reactor experiments and process simulations: yield, biocatalyst yield, and productivity. From the data collected from reactor experiments and process simulations, the following process metrics were calculated:

- Conversion, X—the molar ratio of the product (rhododendrol) and the substrate (betuloside or betuloside and apiosylrhododendrol).
- Productivity (*Pr*)—the mass concentration of rhododendrol per day.
- Biocatalyst yield (*BY*)—the ratio of the rhododendrol mass and the mass of the spent biocatalyst.

## Results and Discussion

This section presents the outcomes of our study of the enzyme-catalyzed production of rhododendrol, an intermediate compound in raspberry ketone production. Our approach utilized the enzymatic hydrolysis of rhododendrol glycosides naturally derived from the inner bark of birch, a renewable resource. The composition of the rhododendrol glycoside mixture varies significantly depending on the origin of the plants, and different proportions of betuloside and apiosylrhododendrin were used in this study. The product of the reaction, rhododendrol, is a chiral compound. The chirality of the products is identical to the chirality of the substrates, but it is not decisive for the observed reaction. It has no influence on the kinetics of the enzymes or the mathematical model presented, as the enzymes used in the reactions presented here belong to the hydrolase group and are not enantioselective per se. They hydrolyze glycosidic bonds. Nevertheless, the product was analyzed on a chiral column (Section S6) and the chromatograms of the products obtained by the hydrolysis of two glycoside mixtures with calculated enantiomeric excesses are shown in Figures S4 and S5.

To evaluate the feasibility of this process, we conducted a comprehensive analysis. We developed a mathematical model based on studies of enzyme kinetics and operational stability, focusing, in particular, on the hydrolysis of betuloside catalyzed by β-glucosidase. This model was validated in batch, repetitive batch, and ultrafiltration membrane reactors.

Furthermore, RAPIDASE showed significant activity for the hydrolysis of apiosylrhododendrin after screening nearly 50 enzymes. The corresponding model was validated in a batch reactor. The optimization based on a mathematical model provided crucial insights for the adjustment of the input parameters, thereby increasing the productivity and achieving the desired results. For the validation of the model and estimated kinetic parameters, the decision was to use the mixture with highest ratio in favor of betuloside and vice versa.

### Hydrolysis of Betuloside

β-Glucosidase-catalyzed hydrolysis of rhododendrol glycosides involves the enzymatic cleavage of glycosidic bonds, resulting in the release of rhododendrol and glucose. Enzymes such as β-glucosidase facilitate this process by breaking the bond between the glucose molecule and rhododendrol. This reaction is important for its potential applications in biotechnological processes, including the production of rhododendrol.^26^

In the work of Becker et al., β-glucosidase from almonds was used for the hydrolysis of apiosylrhododendrin and arabinosylrhododendrin, directing our research to the use of the proposed enzyme.^18^ The rhododendrol glycoside mixture used in this study contained betuloside and apiosylrhododendrin. At the beginning of the research, the analytical results showed that the β-glucosidase can only hydrolyze betuloside (Figure S2). In the absence of the enzyme for the hydrolysis of apiosylrhododendrin, the reaction of betuloside hydrolysis was optimized. Prior to the kinetic tests, it was important to determine the optimal reaction conditions (pH and temperature). The activity and stability of the enzyme were examined using the spectrophotometric test as described in the Experimental Section. Optimal conditions were determined to be at 40 °C and potassium phosphate buffer pH 6 (Figure S6), which is consistent with previously published data for β-glucosidase from almonds.^27,28^

The initial reaction rate method was used to determine the β-glucosidase kinetics. The influence of the betuloside concentration on the initial reaction rate was determined, as was the influence of the products (rhododendrol and glucose). The kinetics of this reaction was described by the single-substrate Michaelis–Menten equation (Table 1, eq 4) with competitive inhibition by rhododendrol (*K*_i1_^rhododendrol^) and glucose (*K*_i1_^glucose^) (Table 1 and Figure S9). From the estimated parameters (Table 1), it can be concluded that rhododendrol inhibits β-glucosidase more strongly than glucose. The literature reports that the use of β-glucosidase in biomass hydrolysis and biofuel production is limited due to its strong inhibition by glucose.^29^ Inhibition by apiosylrhododendrin, which is present in the rhododendrol glycoside mixture, was not detected (data not shown). The inactivation constant, *k*_d_, was described by second-order kinetics (Table 1, eq 11), and the inactivation constants were estimated from the reactor experiments.

**Table 1:** Estimated Kinetic Parameter, Kinetic Model, and Reactor Models for Rhododendrol Glycoside Hydrolysis Catalyzed by β-Glucosidase from Almonds

Kinetic Model
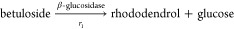 3
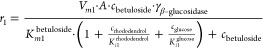 4

parameter	unit	value
*V*_m_	U mg^–1^	8.696 ± 0.328
*K*_m1_^betuloside^	mM	26.701 ± 3.764
*K*_i1_^rhododendrol^	mM	7.135 ± 2.204
*K*_i1_^glucose^	mM	65.493 ± 17.186

Reactor Models	
batch	ultrafiltration membrane
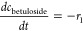 5	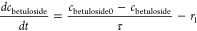 8
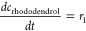 6	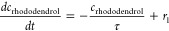 9
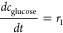 7	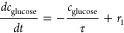 10

Inactivation Model
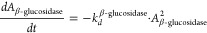 11

Although this process was a part of two patents^30,31^ that were abandoned, there is little data in the literature on the optimization of enzymatically catalyzed rhododendrol glycosides, which makes this research suitable for a detailed insight into raspberry ketone production from renewable resources.

To validate the proposed mathematical model (Table 1), which was developed based on kinetic studies, three experiments were conducted: in a repetitive batch reactor, a batch reactor, and an ultrafiltration membrane reactor.

**Figure 2:**
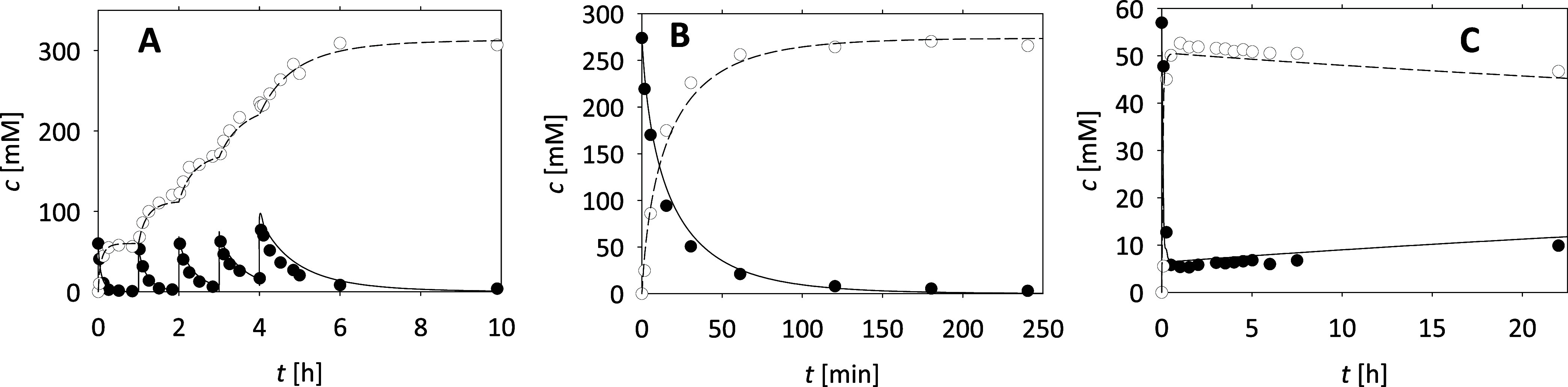
Hydrolysis of crude betuloside (*T* = 40 °C; 100 mM phosphate buffer, pH 6). Correlation of experimental data and model through changes in the concentration of betuloside and rhododendrol over time in (A) repetitive batch reactor (*c*_betuloside_ = 5 × 62.3 mM, γ_β-Glu_ = 4 mg mL^–1^), (B) batch reactor with a higher substrate concentration (*c*_betuloside_ = 275.0 mM, γ_β-Glu_ = 4 mg mL^–1^), and (C) ultrafiltration membrane reaction (*c*_betuloside_ = 62.3 mM, γ_β-Glu_ = 4 mg mL^–1^, *q*_v_ = 180 μL min^–1^). Legend: ● betuloside concentration in experiment, ○ rhododendrol concentration in experiment, – – – betuloside concentration by model, - - - rhododendrol concentration by model.

Initially, a repetitive batch (Figure 2A) was carried out in which a lower concentration of the reactant, betuloside, was added to the reactor five times, each subsequent addition taking place after the previous concentration was fully depleted. This way of performing process is suitable for evaluating the stability of the enzyme over long-term use. As illustrated in Figure 2A, complete conversion of betuloside was achieved within 700 min, resulting in the formation of approximately 300 mM rhododendrol. The proposed model demonstrated a high correlation with the experimental data (*R*^2^ = 0.99, σ = 3.8).

After good results were obtained with the first reaction, albeit with a productivity of 204 g L^–1^ day^–1^, the second reaction was done in a batch reactor with a high initial substrate concentration (275 mM, Figure 2B). Complete conversion was achieved within 2 h, indicating that high substrate concentrations do not adversely affect the reaction rate of betuloside hydrolysis catalyzed by β-glucosidase from almonds. This result is particularly advantageous, as this method is much simpler than the repetitive batch approach and can reach higher productivity (528 g L^–1^ day^–1^). The good correlation between the simulation and the experimental data (*R*^2^ = 0.99, σ = 10.2) suggests that the proposed mathematical model accurately describes the reaction in the batch reactor (Figure 2B).

For final validation, the hydrolysis of betuloside catalyzed by β-glucosidase from almonds was carried out in an ultrafiltration membrane reactor with a flow rate of the reaction mixture of 180 μL min^–1^, corresponding to a retention time of 55.56 min (Figure 2C). The steady-state conversion was approximately 93%, limited by the short retention time and insufficient enzyme concentration. After 3 h, a slight decrease in product concentration and an increase in substrate concentration were observed, which is due to the deactivation of the enzyme. To keep the substrate and product concentrations constant at steady state, the enzyme concentration in the reactor must be higher or residence time should be gradually increased.^32^ The proposed model accurately described the experimental data (*R*^2^ = 0.95, σ = 8.3) (Figure 2C).

The estimated inactivation constants and productivity of each reaction performed to validate the developed mathematical models for betuloside hydrolysis catalyzed by β-glucosidase from almonds are shown in Table 2. The estimated inactivation constant for the UFMR was lower than the ones obtained in BR (Table 2), from which it can be concluded that the membrane stabilizes the enzyme, i.e., the enzyme may have been adsorbed to the membrane, which contributed to its increased stability.^33^ The lower value of productivity in UFMR was the result of lower initial substrate concentration due to the viscosity of rhododendrol glycoside solution as described in the Experimental Section. Despite the above, the conducted experiment showed that it is possible to carry out this reaction in flow mode, which can greatly contribute to the process intensification.^34^

**Table 2:** Estimated Inactivation Constants and Productivity for Rhododendrol Glycoside Hydrolysis Catalyzed by β-Glucosidase from Almonds in Different Reactors

reactor	*k*_d_ [min^–1^]	*Pr* [g L^–1^ day^–1^]
batch	2.71 × 10^–3^	528
repetitive batch	2.79 × 10^–3^	204
ultrafiltration membrane	0.91 × 10^–3^	201–225

### Hydrolysis of Betuloside and Apiosylrhododendrin

In order to achieve better utilization of the process, it was crucial to find the enzyme that can hydrolyze apiosylrhododendrin. For this purpose, almost 50 enzymes were screened (see Table S1). Detailed method for enzyme screening can be found in Section S5. Potocka et al. presented that the enzymatic hydrolysis of diglycosides (e.g., apiosylrhododendrin) may generally proceed by a mechanism that in a sequential mode initially cleaves the intersugar bond between the terminal sugar (in this case apiose) and β-d-glucoside (which is betuloside in this research). In the second step, a β-glucosidase catalyzes the hydrolysis of the resulting β-d-glucoside (betuloside) and releases the corresponding aglycone (rhododendrol) and glucose. They also proposed a series of enzymes and enzyme preparations for the hydrolysis of diglycosides, some of which were included in the screening process.^22^ These enzyme preparations are important for flavor development in the fermentation of wine and black tea. The reaction mechanism was investigated in a paper by Mastihuba et al.^35^ RAPIDASE showed the highest activity among the investigated enzymes. The activity and stability of the enzyme under different conditions (pH and temperature) in potassium phosphate buffer were examined using the spectrophotometric test described in the Experimental Section. *T* = 40 °C and potassium phosphate buffer pH 6 were chosen as compromise conditions (Figures S7 and S8).

As RAPIDASE is, as already mentioned, a mixture of β-glucosidase and polygalacturonase, the initial reaction rate method was used to determine the kinetics of both enzymes. The influence of the betuloside concentration on the initial reaction rate was determined for β-glucosidase, and the influence of the apiosylrhododendrin concentration was determined for polygalacturonase. The influence of all products was also examined as well as the influence of betuloside on polygalacturonase and of apiosylrhododendrin on β-glucosidase. The kinetics of this reaction was described by the single-substrate Michaelis–Menten equation (eqs 3, 13, and 15) with competitive products inhibition (Figures S10 and S11). The kinetic model, estimated kinetic parameters, and reactor models are shown in Table 3, and it can be concluded that betuloside (*K*_i2_^betuloside^), rhododendrol (*K*_i2_^rhododendrol^), and glucose (*K*_i2_^glucose^) inhibit polygalacturonase, including substrate inhibition by apiosylrhododendrin (*K*_is_^apiosylrhododendrin^). The inhibition constant for betuloside (*K*_i2_^betuloside^) was estimated from experimental data from the reactor. β-Glucosidase is significantly inhibited by rhododendrol (*K*_i3_^rhododendrol^) and glucose (*K*_i3_^apiosylrhododendrin^). Inhibition of β-glucosidase by apiose and apiosylrhododendrin was not detected (data not shown).

**Table 3:** Estimated Kinetic Parameters, Kinetic Models, and Reactor Models for Rhododendrol Glycoside Hydrolysis Catalyzed by RAPIDASE

Kinetic Models
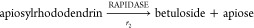 12
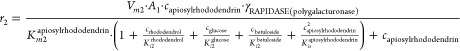 13
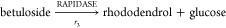 14
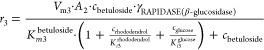 15

parameter	unit	value	parameter	unit	value
apiosylrhododendrin hydrolysis			betuloside hydrolysis		
*V*_m2_	U mg^–1^	0.183 ± 0.060	*V*_m3_	U mg^–1^	0.118 ± 0.007
*K*_m2_^apiosylrhododendrin^	mM	39.070 ± 18.169	*K*_m3_^betuloside^	mM	117.584 ± 32.485
*K*_i2_^rhododendrol^	mM	6.658 ± 0.821	*K*_i3_^rhododendrol^	mM	3.566 ± 0.732
*K*_i2_^glucose^	mM	9.450 ± 3.178	*K*_i3_^glucose^	mM	2.646 ± 0.704
*K*_i2_^betuloside^	mM	3.001 ± 1.156			
*K*_is_^apiosylrhododendrin^	mM	80.764 ± 45.685			

Reactor Models		
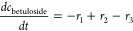 16	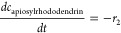 17	
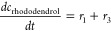 18	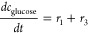 19	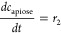 20

To validate the developed mathematical model and estimated kinetic parameters, a total of four reactions were carried out: 1. RAPIDASE + batch of rhododendrol glycosides with a higher apiosylrhododendrin ratio, 2. RAPIDASE + rhododendrol glycosides with a higher betuloside ratio, 3. RAPIDASE + β-glucosidase from almonds + batch of rhododendrol glycosides with a higher apiosylrhododendrin ratio, 4. RAPIDASE and β-glucosidase from almonds using a batch of rhododendrol glycosides with a higher betuloside ratio.

**Figure 3:**
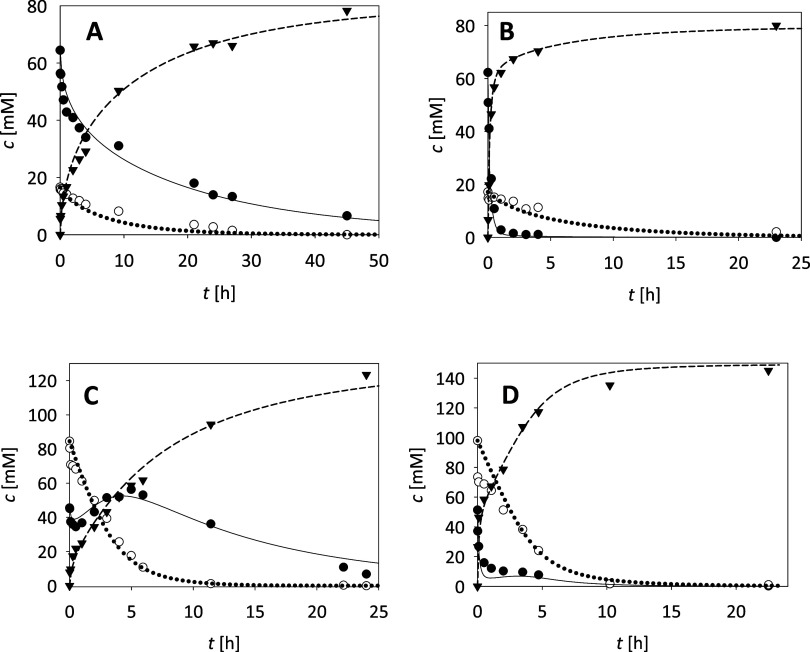
Hydrolysis of betuloside and apiosylrhododendrin mixture (*T* = 40 °C; 100 mM phosphate buffer, pH 6). Correlation of experimental data and model through changes in the concentration of betuloside, apiosylrhododendrin, and rhododendrol over time in a batch reactor: (A) *c*_betuloside_ = 64.5 mM, *c*_apiosylrhododendrin_ = 16.6 mM, γ_RAPIDASE_ = 20.0 mg mL^–1^, (B) *c*_betuloside_ = 62.3 mM, *c*_apiosylrhododendrin_ = 17.2 mM, γ_RAPIDASE_ = 20.0 mg mL^–1^, γ_β-Glu_ = 2.0 mg mL^–1^, (C) *c*_betuloside_ = 45.5 mM, *c*_apiosylrhododendrin_ = 84.6 mM, γ_RAPIDASE_ = 100.0 mg mL^–1^, (D) *c*_betuloside_ = 51.3 mM, *c*_apiosylrhododendrin_ = 98.0 mM, γ_RAPIDASE_ = 100.0 mg mL^–1^, γ_β-Glu_ = 2.0 mg mL^–1^. Legend: ● betuloside concentration in experiment, ○ apiosylrhododendrin concentration in experiment, rhododendrol concentration in experiment, – – – betuloside concentration by model, **·····** apiosylrhododendrin concentration by model, - - - rhododendrol concentration by model.

Numerous RAPIDASE preparations from the DMS Oenobrands portfolio are intended for the processing of fruits, vegetables, and wine.^36^ Ortega et al. carried out kinetic and thermal behavior tests for the polygalacturonase contained in RAPIDASE C80.^37^ As in our study, the kinetics was also described using the single-substrate Michaelis–Menten kinetics. By investigating the thermal stability, they found that the two-fraction inactivation model can describe the decrease in enzyme activity with the highest accuracy. This is consistent with this study, in which the biexponential model^38^ was used to model the operational stability of RAPIDASE (Table 4) in the experiments shown in Figure 3.

**Table 4:** Estimated Inactivation Constants for Rhododendrol Glycoside Hydrolysis Catalyzed by β-Glucosidase from Almonds and Polygalacturonase from RAPIDASE in a Batch Reactor

Inactivation Model
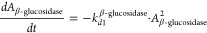 21
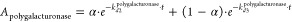 22

parameter	unit	value
*k*_d1_^β-glucosidase^	min^–1^	0.11 × 10^–3^
*k*_d2_^polygalacturonase^	min^–1^	0.09 × 10^–3^
*k*_d3_^polygalacturonase^	min^–1^	9.93 × 10^–3^
α		0.520

In the first reaction where the share of betuloside is higher and only RAPIDASE is applied for catalysis of hydrolysis, (Figure 3A), the reactions of both betuloside and apiosylrhododendrin hydrolysis were slow; after 27 h, full conversion of only apiosylrhododendrin is achieved. Since the reaction of betuloside hydrolysis by β-glucosidase present in the RAPIDASE mixture is slower than apiosylrhododendrin hydrolysis by polygalacturonase and the concentration of betuloside in this reaction is higher than the apiosylrhododendrin, betuloside hydrolysis can be considered as the bottleneck of the process affecting the speed of the reaction overall. The mathematical model was validated, as confirmed by the statistical output (*R*^2^ = 0.99, σ = 3.2). When to the same substrate both RAPIDASE and β-glucosidase from almonds are added (Figure 3B), full conversion of both rhododendrol glycosides was achieved. The proposed model was accurately validated by the final experiment (*R*^2^ = 0.98, σ = 4.8). The reaction rate of apiosylrhododendrin hydrolysis was the same in both reactions. In the case of lower betuloside concentration in the reaction mixture, when only RAPIDASE is used, it was noticeable that after 30 min, the concentration of betuloside increases due to liberation of the compound in apiosylrhododendrin hydrolysis (Figure 3C). The *K*_m_ value of apiosylrhododendrin for polygalacturonase is 3 times lower than the one for β-glucosidase (present in RAPIDASE) (Table 3) which makes the reaction of apiosylrhododendrin hydrolysis preferable. Although betuloside hydrolysis occurs throughout, the decrease in betuloside concentration was only evident when most of the apiosylrhododendrin was converted. This reaction also confirms the developed model (*R*^2^ = 0.99, σ = 5.2). This effect was significantly lower when both RADPIDASE and β-glucosidase from almonds were used, as the reaction rate of betuloside hydrolysis by β-glucosidase from almonds is higher (Figure 3D). The proposed model was accurately validated by the final experiment (*R*^2^ = 0.97, σ = 13.7). The productivity of each experiment is not comparable because substrate concentrations are not the same and not the same enzyme concentrations were used. In the reactions with higher betuloside concentrations, 20 mg mL^–1^ RAPIDASE was used. Higher productivity was achieved when both enzymes were used (*Pr* = 13.9 g L^–1^ day^–1^) compared to the experiment with only RAPIDASE (*Pr* = 6.9 g L^–1^ day^–1^). In the reactions with lower betuloside concentration, 100 mg mL^–1^ RAPIDASE was used. Higher productivity was achieved when both enzymes were used (*Pr* = 25.7 g L^–1^ day^–1^) compared to the experiment with only RAPIDASE (*Pr* = 20.4 g L^–1^ day^–1^).

The estimated inactivation constants for β-glucosidase and polygalacturonase were the same in all experiments (Table 4). It can be seen that β-glucosidase was significantly more stable in this series of experiments (*k*_d1_^β-glucosidase^, up to 25-fold, Table 4) than in the reactions in which only betuloside hydrolysis was performed (Table 2). This can be attributed to the stabilization of the enzyme due to the presence of a high protein concentration in the reactor, as these experiments were carried out with a considerable amount of the RAPIDASE enzyme preparation.^39^

The development of β-apiosidases as enzymes that can catalyze diglycosides was of significance in these fields.^40−44^ The optimization of these processes becomes particularly important when considering the broader applications of these low-cost enzymes beyond the food and wine industries. Their potential lies particularly in the nutraceutical and pharmaceutical industries, where their incorporation could offer substantial benefits. Due to the low activity of RAPIDASE, which can be marked as the major limitation of the research, optimizing the application of the enzyme is critical to maximizing their efficacy and cost-effectiveness in applications such as those described in this work.

### Model-Based Simulations

The proportion of betuloside and apiosylrhododendrin in the mixture of rhododendrol glycosides can vary significantly following extraction from birch bark. Since the hydrolysis of these two glycosides is catalyzed by distinct enzymes (β-glucosidase and RAPIDASE), it is crucial to predict the required enzyme amounts to ensure satisfactory and uniform process metrics regardless of the composition of the extracted mixture. Utilizing the validated model, simulations were conducted with various glycoside mixtures. The proportion of betuloside ranged from 0.2 to 0.9 (step 0.1). For each glycoside mixture, simulations were performed at different initial enzyme concentrations, with an initial glycoside concentration of 300 mM. The concentration of β-glucosidase was varied between 2 and 4 mg mL^–1^ (step 0.2 mg mL^–1^), and RAPIDASE was varied between 20 and 200 mg mL^–1^ (step 20 mg mL^–1^).

Examples of simulations for one combination of enzymes and for three different glycoside mixtures are provided in Figure S12.

**Figure 4:**
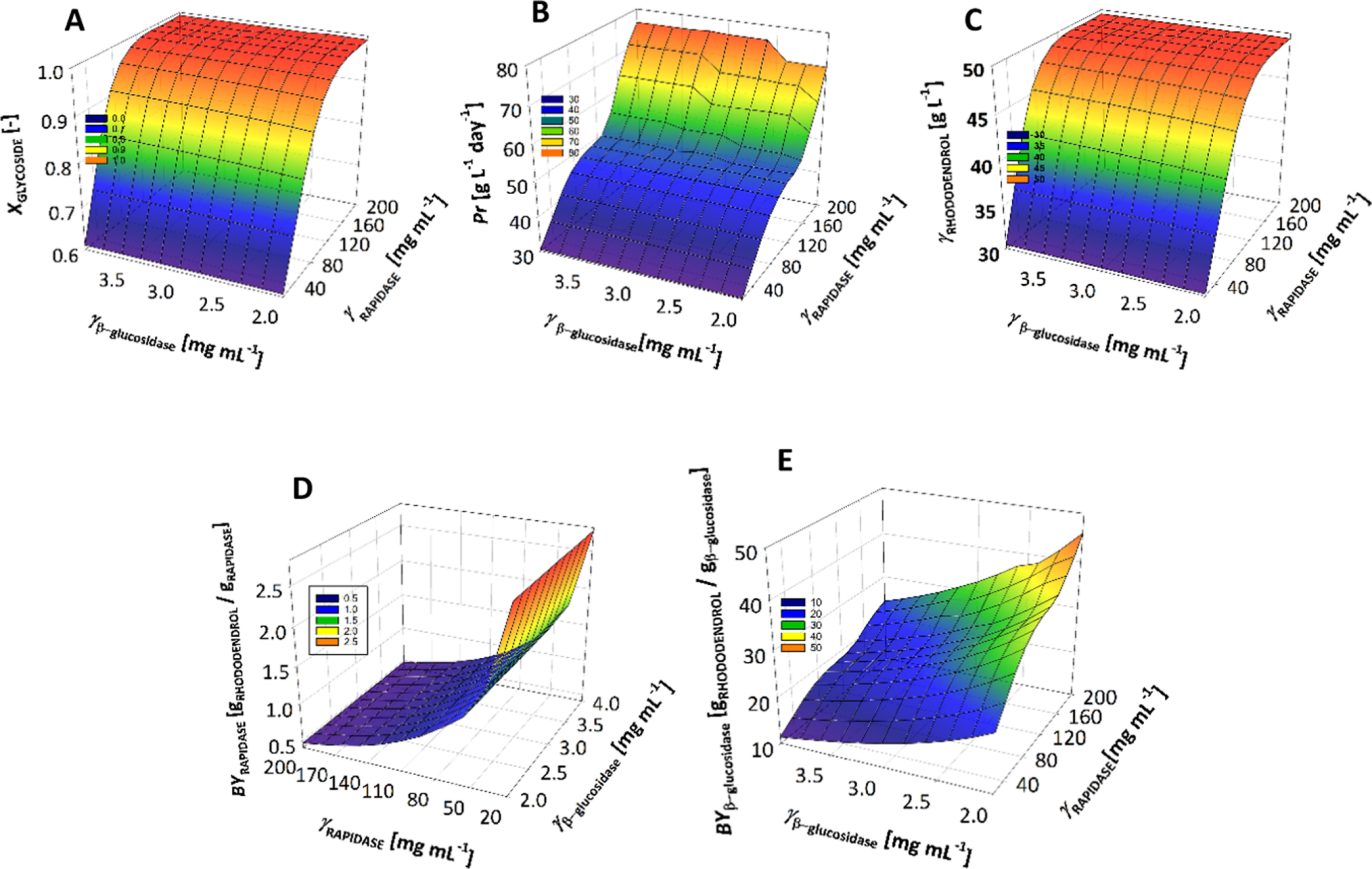
Dependence of process metrics on enzyme concentrations for a mixture of rhododendrol glycosides (*x*_betuloside_ = 0.5, *c*_rhod. glyc_. = 300 mM). The results for each enzyme’s initial concentration were taken at the time when *X*_rhod.glyc_. = 99%. (A) Conversion of rhododendrol glycosides, (B) productivity, (C) final rhododendrol concentration (in g L^–1^), (D) biocatalyst yield for RAPIDASE, and (E) biocatalyst yield for β-glucosidase from almonds.

For each enzyme combination, the result was taken when 99% conversion was achieved or after 24 h. Based on these results, conversion, productivity, final product concentration, and biocatalyst yield for each enzyme were calculated. An example of the dependence of process metrics on enzyme concentration for a glycoside mixture with a betuloside share of 0.5 is shown in Figure 4.

The results indicated that the productivity, the conversion of glycosides, and the final concentration of rhododendrol were significantly dependent on the concentration of RAPIDASE. RAPIDASE has a total glycosidase content of 1–10%, the specific proportion of the polygalacturonase enzyme being unknown. Consequently, the specific activity of RAPIDASE in the hydrolysis of apiosylrhododendrin is very low (Table 3). Additionally, RAPIDASE is significantly inhibited by reaction products, such as glucose and rhododendrol (Table 3). Therefore, if the amount of apiosylrhododendrin in the mixture is high, the required amount of RAPIDASE enzyme must be carefully determined.

**Figure 5:**
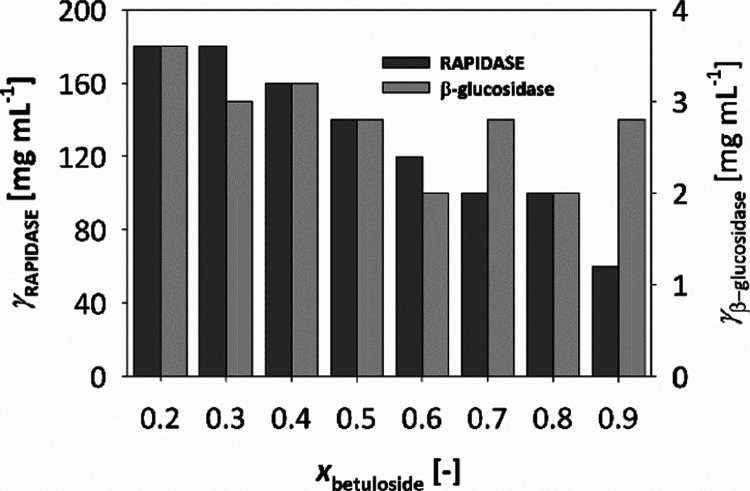
Optimal initial concentrations of the enzymes RAPIDASE and β-glucosidase for the hydrolysis of rhododendrol glycoside mixtures (*c*_0_ = 300 mM), ensuring a substrate conversion of 99% within 24 h determined by simulation.

Based on this analysis, the optimal enzyme concentration for each glycoside mixture (with proportions between 0.2 and 0.9) was identified as the one that achieves 99% conversion at the lowest concentration of RAPIDASE (Figure 5).

The amount of RAPIDASE required increases significantly (from 60 to 180 mg mL^–1^) with an increase in the proportion of apiosylrhododendrin. Considering the higher stability of the RAPIDASE enzyme, its remaining activity is about 45%, compared to 35% for β-glucosidase. By subsequently separating the enzyme from the mixture through ultrafiltration, these remaining activities can be reused, thereby significantly enhancing the process’s economic efficiency.^45^ The productivity of this process ranged from 48 to 58 g of rhododendrol L^–1^ day^–1^.

Although mixtures with a high content of apiosylrhododendrin require significantly higher amounts of RAPIDASE, it is important to note that such mixtures also yield more apiose monosaccharides (4–35 g L^–1^, Figure S13). This compound is rarely found in nature as a “free sugar”, making its market price quite high. It has potential applications in the synthesis of apiosides and apioglucosides, which could be useful in the pharmaceutical industry.^22^ The amount of glucose produced in these processes is about 52 g L^–1^, which can also be used as a substrate for generating value-added products.^46^

This demonstrates how model-based simulation can speed up the decision-making process for adjusting the input parameters when the composition of the input components is uneven and ensures a uniform end result.

## Conclusions

In this study, we successfully optimized the biocatalytic production of rhododendrol from natural substrates using a combination of β-glucosidase and polygalacturonase enzymes. Through extensive screening, we identified RAPIDASE and β-glucosidase from almonds as the most effective biocatalysts for the hydrolysis of rhododendrol glycosides. Systematic kinetic studies revealed the optimal conditions for enzymatic activity, including temperature, pH, and substrate concentrations.

Mathematical modeling and simulations provided valuable insights into the reaction mechanisms, refining the process parameters and enhancing rhododendrol yield. The compliance of the experimental data with the model-based simulations demonstrated the feasibility of this biocatalytic approach for sustainable rhododendrol production.

This research highlights the potential of utilizing natural resources for the efficient and environmentally friendly synthesis of valuable compounds, such as rhododendrol. Future studies could investigate the scaling up of the process and the application of additional biocatalysts to further improve the productivity and cost efficiency.

Overall, our work contributes to the field of biocatalysis and emphasizes the importance of optimizing enzymatic reactions for industrial applications. The progress made will enable more sustainable production methods in the nutraceutical, pharmaceutical, and cosmetics industries.

## Supporting Information Available

Additional experimental details, methods, and reaction schemes; chromatograms, calibration curves, and experimental results regarding activity and stability of the enzymes; their kinetics; and model-based simulations (PDF)
