# Supplementary material for: Optimization of Biocatalytic Rhododendrol Production from Biogenic Rhododendrol Glycosides
Source: ACS Sustain Chem Eng. 2024 Oct 18;12(44):16329–39. doi: 10.1021/acssuschemeng.4c05889 (PMC11539071; doi:10.1021/acssuschemeng.4c05889)
Supplement: Supplementary file 1 — sc4c05889_si_001.pdf [file sc4c05889_si_001.pdf]

SUPPLEMENTARY INFORMATION

Optimization of biocatalytic rhododendrol production from biogenic rhododendrol glycosides

Emerik Leaković†, Karsten Siems‡, Michel Feussi Tala‡, Antonia Habazin†, Zvjezdana Findrik Blažević†, Ana Vrsalović Presečki†*

†University of Zagreb Faculty of Chemical Engineering and Technology, Trg Marka Marulića 19, HR-10000 Zagreb, Croatia

‡AnalytiCon Discovery GmbH, Hermannswerder 17, 14473 Potsdam, Germany

***Corresponding author:**

Ana Vrsalović Presečki

tel.: +385 1 4597 157

e-mail: avrsalov@fkit.hr

Number of pages: 14

Number of figures: 15

Number of tables: 1

Table of contents

1. Supplementary figures S3
    1.1. Analytics S3
    1.2. Activity and stability of the enzymes S5
    1.3. Kinetics S7
    1.4. Model based simulations S8
    1.5. Reaction schemes S9
2. Supplementary tables S10
3. Reagent preparation S11
4. Validation of PGA-DNS method for measuring polygalacturonase activity S12
5. Enzyme screening method for apiosylrhododendrol hydrolysis S13
6. HPLC method for enantiomers quantification S14

1. Supplementary figures

1.1. Analytics

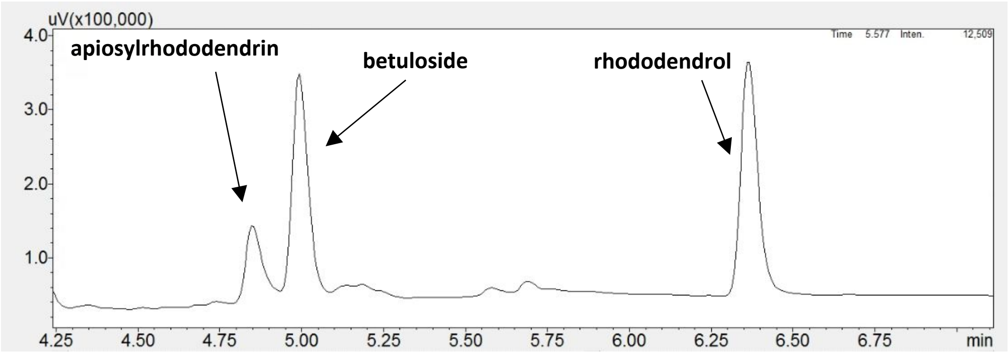

Figure S1. Chromatogram showing peeks of apiosylrhododendrin, betuloside and rhododendrol.

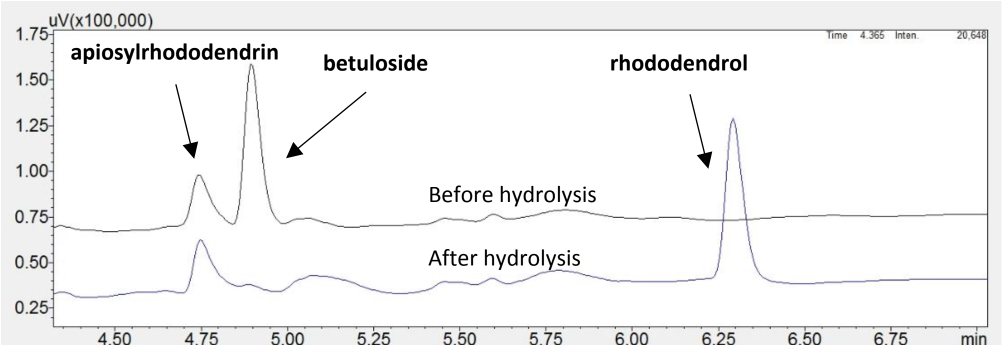

Figure S2. Chromatogram showing peeks of apiosylrhododendrin, betuloside and rhododendrol before and after hydrolysis catalyzed by β – glucosidase from almond.

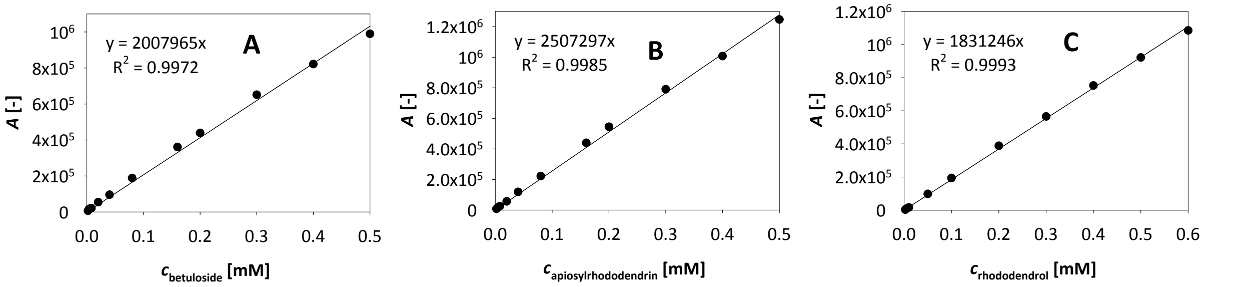

Figure S3. Calibration curves for HPLC measurements. Peek area dependence on **A)** betuloside concentration ranging from 0.002 mM to 0.5 mM, **B)** apiosylrhododendrin concentration ranging from 0.002 mM to 0.5 mM and **C)** rhododendrol concentration ranging from 0.0025 mM to 0.6 mM.

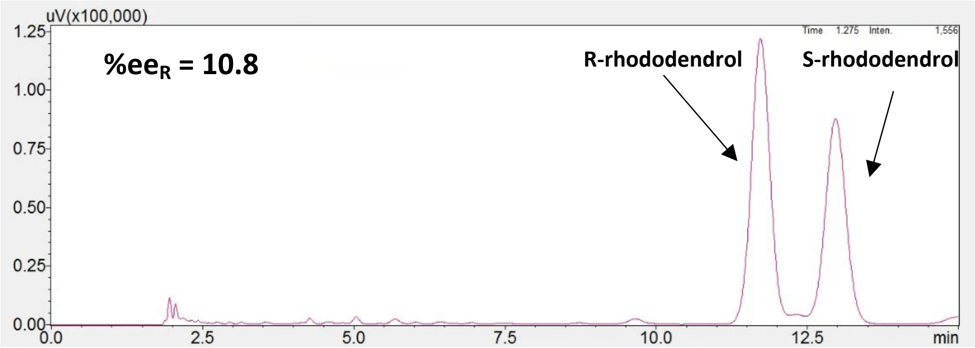

Figure S4. Chromatogram showing peeks of R-rhododendrol and S-rhododendrol after hydrolysis of rhododendrol glycosides (mixture 1: $\omega_{betuloside}$ = 74.0 %, $\omega_{apiosylrhododendrin}$ = 26.0 %).

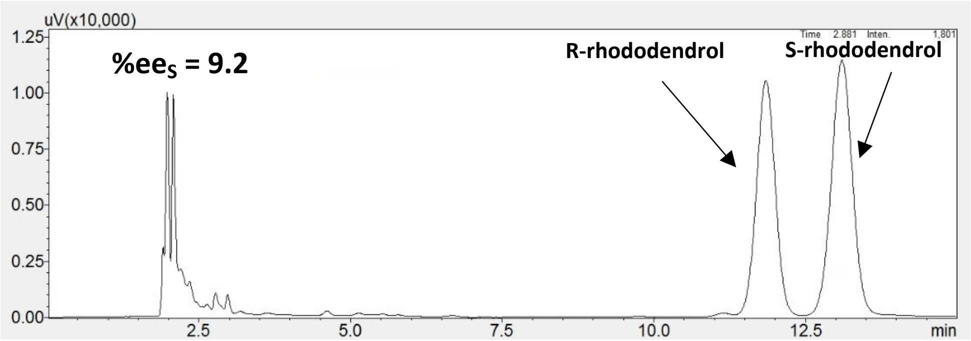

Figure S5. Chromatogram showing peeks of R-rhododendrol and S-rhododendrol after hydrolysis of rhododendrol glycosides (mixture 2: $\omega_{betuloside}$ = 27.5 %, $\omega_{apiosylrhododendrin}$ = 72.5 %).

1.2. Activity and stability of the enzymes

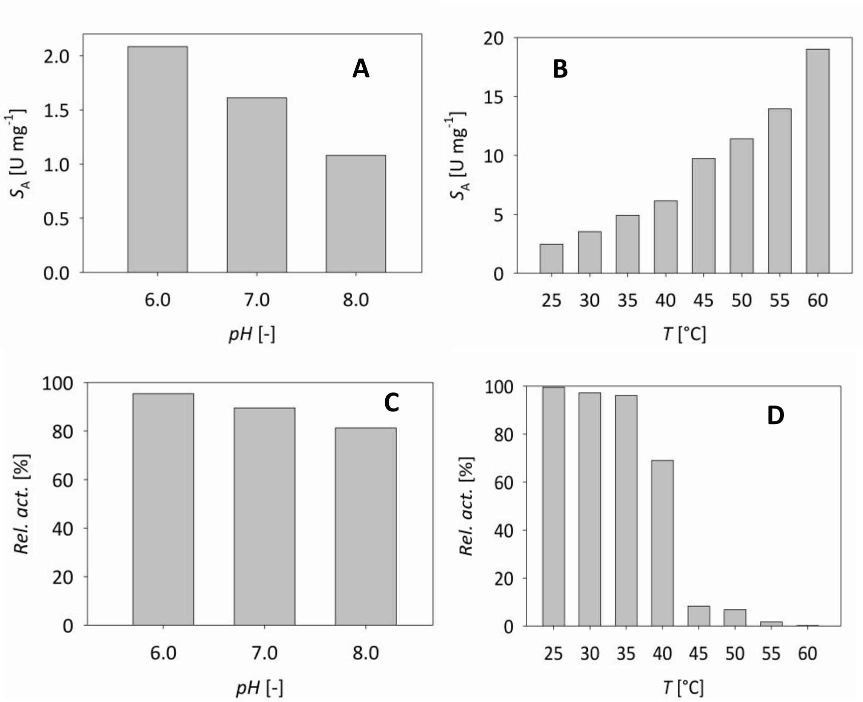

Figure S6. Specific activity of β–glucosidase from almonds in 0.1 M potassium phosphate buffer, $c_{betuloside}$ = 0.025 M, $\gamma_{\beta\text{–glucosidase}}$ = 0.05 mg mL^{-1}, **A)** vs. pH at T = 25 °C and **B)** vs. temperature at pH 6. Residual activity of β–glucosidase from almonds in 0.1 M potassium phosphate buffer 24 h incubation, p-NPG test, **C)** vs. pH at $T_{incubation}$= 25 °C and **D)** vs. temperature at pH 6.

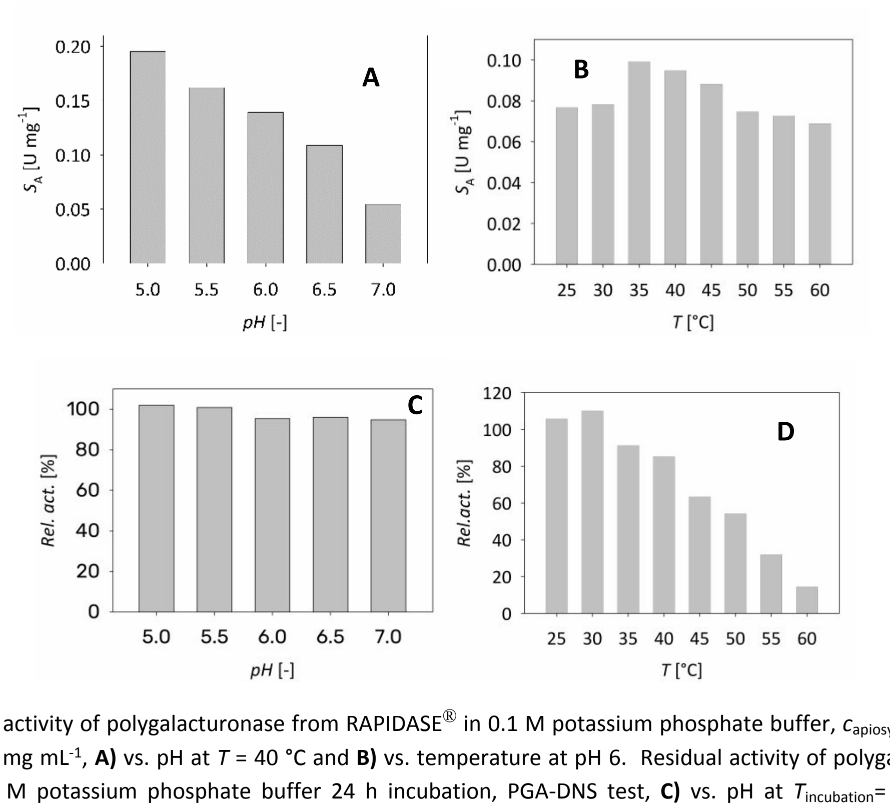

Figure S7. Specific activity of polygalacturonase from RAPIDASE® in 0.1 M potassium phosphate buffer, $c_{apiosylrhododendrin}$ = 0.025 M, $\gamma_{RAPIDASE®}$ = 5.0 mg mL^{-1}, **A)** vs. pH at T = 40 °C and **B)** vs. temperature at pH 6. Residual activity of polygalacturonase from RAPIDASE® in 0.1 M potassium phosphate buffer 24 h incubation, PGA-DNS test, **C)** vs. pH at $T_{incubation}$= 25 °C and **D)** vs. temperature at pH 6.

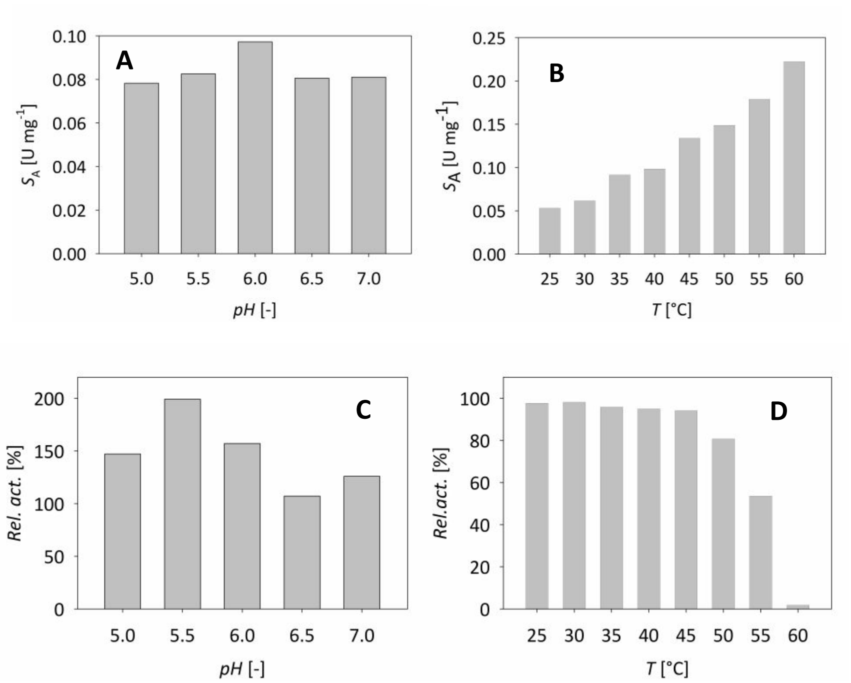

Figure S8. Specific activity of β–glucosidase from RAPIDASE® in 0.1 M potassium phosphate buffer, $c_{apiosylrhododendrin}$ = 0.025 M, $\gamma_{RAPIDASE®}$ = 5.0 mg mL^{-1}, **A)** vs. pH at T = 40 °C and **B)** vs. temperature at pH 6. Residual activity of β–glucosidase from RAPIDASE® in 0.1 M potassium phosphate buffer 24 h incubation, PGA-DNS test, **C)** vs. pH at $T_{incubation}$= 25 °C and **D)** vs. temperature at pH 6.

1.3. Kinetics

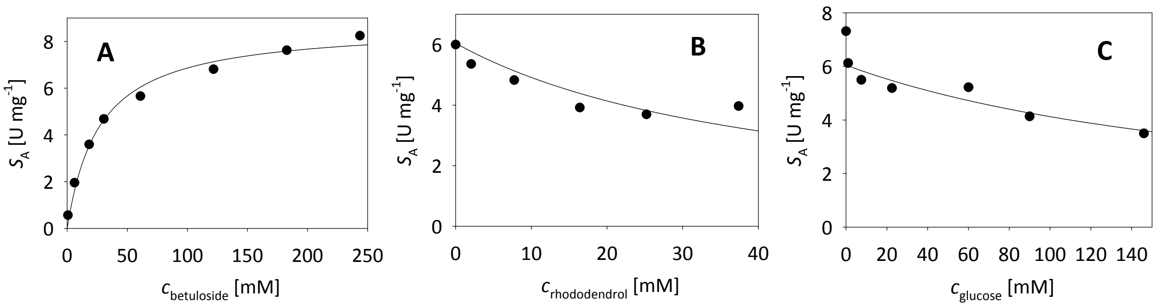

Figure S9. Kinetics of the betuloside hydrolysis catalyzed by β–glucosidase from almonds in 0.1 M potassium phosphate buffer pH 6, $\gamma_{\beta\text{–glucosidase}}$ = 0.05 mg mL^{-1}, T = 40 °C. The dependence of the initial reaction rate on **A)** substrate concentration **B)** product concentration (rhododendrol), $c_{betuloside}$ = 60.9 mM and **C)** product concentration (glucose), $c_{betuloside}$ = 60.9 mM. Legend: ● experiment, —— model.

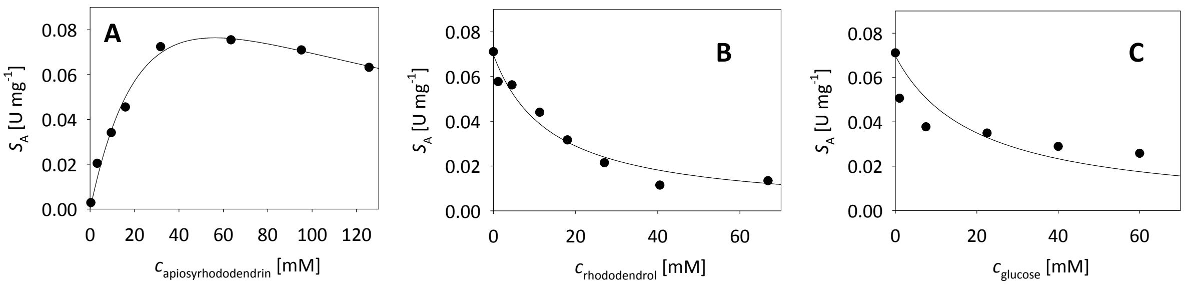

Figure S10. Kinetics of the apiosylrhododendrin hydrolysis catalyzed by polygalacturonase from RAPIDASE® in 0.1 M potassium phosphate buffer pH 6, $\gamma_{RAPIDASE®}$ = 5.0 mg mL^{-1}, T = 40 °C. The dependence of the initial reaction rate on **A)** substrate concentration **B)** product concentration (rhododendrol), $c_{betuloside}$ = 31.7 mM and **C)** product concentration (glucose), $c_{betuloside}$ = 31.7 mM. Legend: ● experiment, —— model.

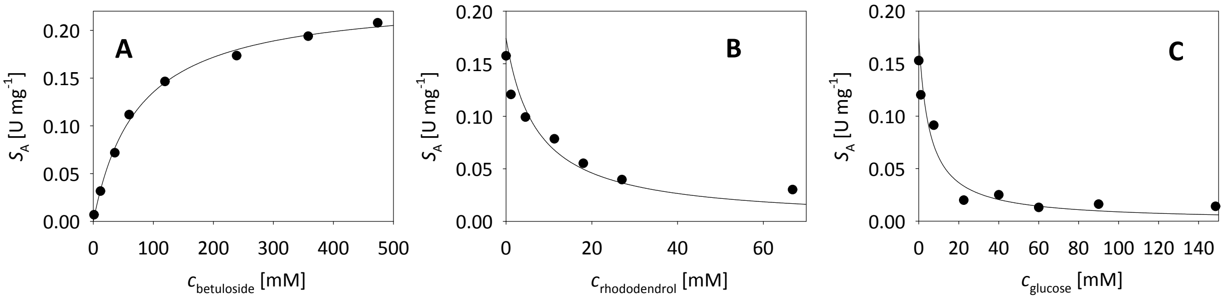

Figure S11. Kinetics of the betuloside hydrolysis catalyzed by β–glucosidase from RAPIDASE® in 0.1 M potassium phosphate buffer pH 6, $\gamma_{RAPIDASE®}$ = 5.0 mg mL^{-1}, T = 40 °C. The dependence of the initial reaction rate on **A)** substrate concentration **B)** product concentration (rhododendrol), $c_{betuloside}$ = 151.1 mM and **C)** product concentration (glucose), $c_{betuloside}$ = 151.1 mM. Legend: ● experiment, —— model.

1.4. Model based simulations

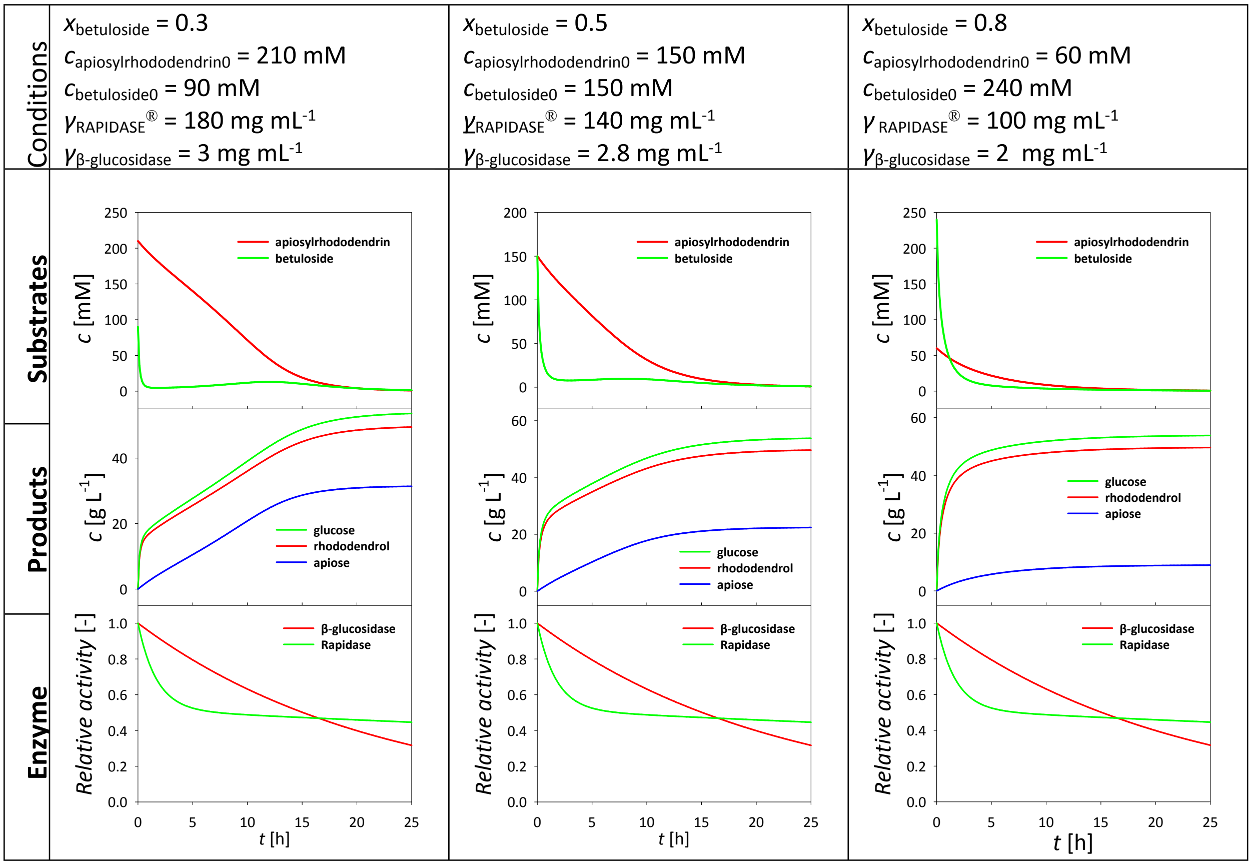

Figure S12. Simulations of rhododendrol glycoside hydrolysis catalysed by RAPIDASE® and *β*-glucosidase.

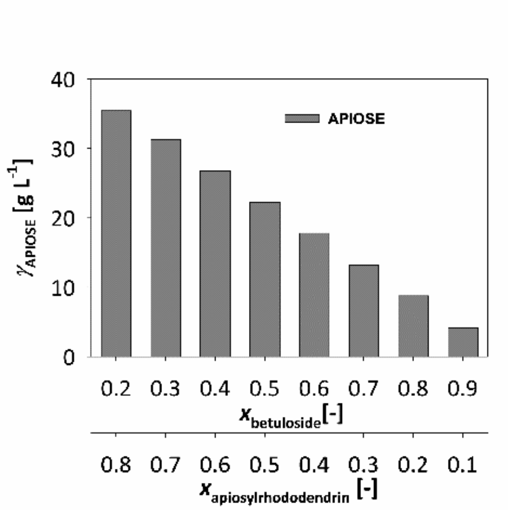

Figure S13. The final concentration of the apiose obtained in the hydrolysis of rhododendrol glycoside mixtures (c_0 = 300 mM) catalysed by optimal concentration of RAPIDASE® and β-glucosidase determined by simulation.

1.5. Reaction schemes

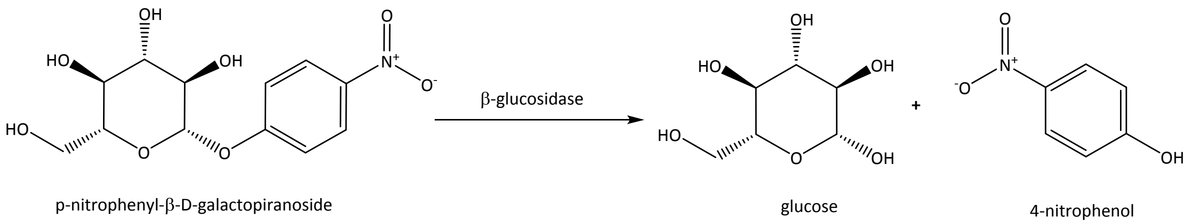

S14. Reaction scheme for β-glucosidase activity assay

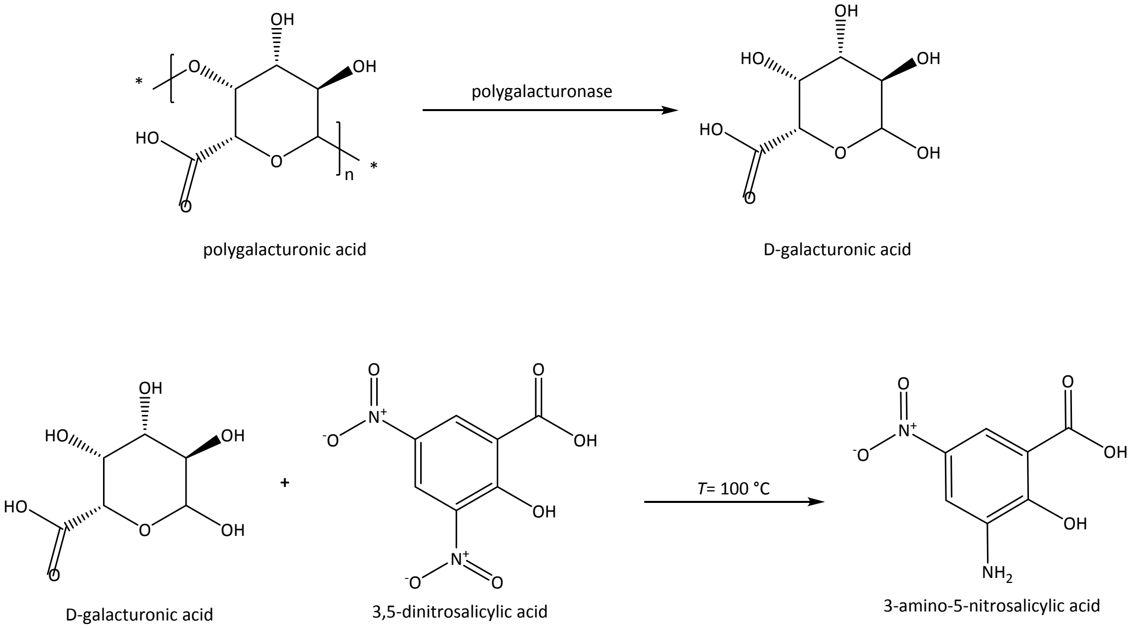

S15. Reactions scheme for PGA-DNS test in polygalacturonase activity assay

2. Supplementary tables

Table S1 List of enzymes and enzymes preparations screened for apiosylrhododendrin hydrolysis with corresponding results expressed as the conversion.

Nr.	Enzyme or enzyme preparation	Information	X [%]	Nr.	Enzyme or enzyme preparation	Information	X [%]
1.	β-glucosidase	Prozomix Limited PRO-BGLUC 001	0	25.	β-glucosidase	Prozomix Limited PRO-BGLUC 025	0
2.	β-glucosidase	Prozomix Limited PRO-BGLUC 002	0	26.	β-glucosidase	Prozomix Limited PRO-BGLUC 026	0
3.	β-glucosidase	Prozomix Limited PRO-BGLUC 003	0	27.	β-glucosidase	Prozomix Limited PRO-BGLUC 027	0
4.	β-glucosidase	Prozomix Limited PRO-BGLUC 004	0	28.	β-glucosidase	Prozomix Limited PRO-BGLUC 028	0
5.	β-glucosidase	Prozomix Limited PRO-BGLUC 005	0	29.	β-glucosidase	Prozomix Limited PRO-BGLUC 029	0
6.	β-glucosidase	Prozomix Limited PRO-BGLUC 006	0	30.	β-glucosidase	Prozomix Limited PRO-E0105	0
7.	β-glucosidase	Prozomix Limited PRO-BGLUC 007	0	31.	β-glucosidase	Prozomix Limited PRO-E0110	0
8.	β-glucosidase	Prozomix Limited PRO-BGLUC 008	0	32.	β-glucosidase	Prozomix Limited PRO-E0121	0
9.	β-glucosidase	Prozomix Limited PRO-BGLUC 009	0	33.	Termamyl®	novozymes	0
10.	β-glucosidase	Prozomix Limited PRO-BGLUC 010	0	34.	Sweetzyme®	novozymes	0
11.	β-glucosidase	Prozomix Limited PRO-BGLUC 011	0	35.	Cellic® CTec2	novozymes	0
12.	β-glucosidase	Prozomix Limited PRO-BGLUC 012	0	36.	glucose oxidase	*Aspergillus niger*, Sigma-Aldrich	0
13.	β-glucosidase	Prozomix Limited PRO-BGLUC 013	0	37.	xylanase	*Aspergillus oryzae*, Sigma-Aldrich	0
14.	β-glucosidase	Prozomix Limited PRO-BGLUC 014	0	38.	pectinase	*Aspergillus niger*, Sigma-Aldrich	0
15.	β-glucosidase	Prozomix Limited PRO-BGLUC 015	0	39.	pectinase	*Aspergillus aculeatus*, Sigma-Aldrich	0
16.	β-glucosidase	Prozomix Limited PRO-BGLUC 016	0	40.	cellulase	*Aspergillus niger*, Sigma-Aldrich	0
17.	β-glucosidase	Prozomix Limited PRO-BGLUC 017	0	41.	NATUZYM® Pectinaze conc.	WeissBiotech	0
18.	β-glucosidase	Prozomix Limited PRO-BGLUC 018	0	42.	DELTAZYM® VR AC-100	WeissBiotech	0
19.	β-glucosidase	Prozomix Limited PRO-BGLUC 019	0	43.	NATUZYM® V-50	WeissBiotech	0
20.	β-glucosidase	Prozomix Limited PRO-BGLUC 020	0	44.	NATUZYM® PC MG	WeissBiotech	0
21.	β-glucosidase	Prozomix Limited PRO-BGLUC 021	0	45.	NATUZYM® arom MG	WeissBiotech	0
22.	β-glucosidase	Prozomix Limited PRO-BGLUC 022	0	46.	Vyscozyme®	novozymes	0
23.	β-glucosidase	Prozomix Limited PRO-BGLUC 023	0	47.	LALLYZYME Beta	Lallemand	80
24.	β-glucosidase	Prozomix Limited PRO-BGLUC 024	0	48.	RAPIDASE® aroma revelation	DSM Oenobrands	98

3. Reagent preparation

Preparation steps of 3,5-dinitrosalicylic acid (DNS) solution 100 mL:

1. Dissolve 30 g of potassium sodium tartrate tetrahydrate in 20 mL distilled water. Add the salt to water gradually.
2. Prepare 2 M sodium hydroxide solution 20 mL.
3. Dissolve 1 g of 3,5-dinitrosalicylic acid in 50 mL of distilled water while the solution is mixed by magnetic stirrer with hot plate at 90-95 ˚C.
4. Add gradually the solution of potassium sodium tartrate tetrahydrate (prepared solution in Step 1) to 3,5-dinitrosalicylic acid solution (prepared solution the Step 3) while the solution is mixed by magnetic stirrer with hot plate at 90-95˚C.
5. Add slowly 2 M sodium hydroxide solution (the solution prepared in step 2) to the solution prepared in step 4 while the solution is mixed by magnetic stirrer with hot plate at 90-95˚C.
6. After the components are completely dissolved, filter the final solution by filter paper.
7. Transfer the solution in dark glass bottles and storage at ambient temperature.

4. Validation of PGA-DNS method for measuring polygalacturonase activity

Solution of 250 mg mL^{-1} of RAPIDASE® was prepared in phosphate potassium buffer pH 6 and was incubated at 40 °C and 900 rpm. The samples for the activity measurements were taken before the incubation and after 24 h. The activity in both samples were measured by two methods:

1. Apiosylrhododendrin hydrolysis assays - the reaction of apiosylrhododendrin hydrolysis was started with the sample taken from the incubated solution in phosphate potassium buffer pH 6 at 40 °C, $c_{\mathrm{apiosylrhododendrin}}$ = 29.8 mM, RAPIDASE® = 5 mg mL^{-1}. Initial reaction rate was determined from the change of rhododendrol concentration determined by HPLC.
2. DNS test

Validation was done in triplets. The residual activity of polygalacturonase in RAPIDASE® after 24 hours was 79.8 ± 3.8 % and 82.8 ± 4.5 % when the PGA-DNS and apiosylrhododendrin hydrolysis assays were used. This indicates that the DNS method can be used to measure polygalacturonase activity.

5. Enzyme screening method for apiosylrhododendrol hydrolysis

Screening process for apiosylrhododendrin hydrolysis was done by performing reaction in the reactor for 24 h. Reaction conditions were:

- T = 40 ° C
- 0.1 M potassium phosphate buffer
- pH = 6
- t =24 h
- 900 rpm
- V_R = 400 mL
- $c_{\text{rhododendrol glycosides}}$ = 10 mg mL^{-1}
- c_{enzyme} = 2 mg mL^{-1},

where T is temperature, t is time and V_R is a reactor volume.

After 24 h a sample from each reaction mixture was analysed via HPLC and from the results the conversion of apiosylrhododendrin was calculated and is showed in table S1, for each enzyme used.

6. HPLC method for enantiomers quantification

The analysis was done at 30 °C on Phenomenex Lux® 5 µm Cellulose-1 (250 x 4.6 mm) LC column equipped with compatible precolumn, at the flow-rate of 1.5 mL min^{-1} by using isocratic flow method. Mobile phase was the mixture of *n*-hexane and isopropanol (9:1, *v*/*v*). The detection was done at 220 nm. The retention time of R-rhododendrol and S-rhododendrol was 11.4 and 12.6 minutes, respectively. Examples of chromatograms are shown in Supplement (Fig. S4 and S5).
